# Adherence to visual field examination in glaucoma patients during the Coronavirus disease 2019 outbreak

**DOI:** 10.1097/MD.0000000000035314

**Published:** 2023-10-06

**Authors:** Pei-Yao Chang, Yu-Han Wang, Jiun-Yi Wang, Jia-Kang Wang

**Affiliations:** a Department of Ophthalmology, Far Eastern Memorial Hospital, Ban-Chiao, New Taipei City, Taiwan; b Department of Healthcare Administration, Asia University, Taichung, Taiwan, Republic of China; c Department of Medicine, National Taiwan University Hospital, Taipei, Taiwan; d Department of Electrical Engineering, Yuan Ze University, Taoyuan City, Taiwan; e Department of Medical Research, China Medical University Hospital, China Medical University, Taichung, Taiwan, Republic of China.

**Keywords:** coronavirus disease 2019 (COVID-19) outbreak, glaucoma, visual field

## Abstract

We described the proportion of adherence to the scheduled visual field (VF) examination and the associated factors in glaucoma patients in a tertiary referral center during the Coronavirus disease 2019 (COVID-19) outbreak in Taiwan. Patients with scheduled VF examinations during May 25th to July 12th, 2021, were retrospectively evaluated. Clinical characteristics including type of glaucoma, intraocular pressure (IOP) at the day of arranging VF examinations, prescriptions of anti-glaucoma medications, non-medical glaucoma treatment, length of glaucoma history, mean deviation (MD) of VF defect, and announcement of glaucoma progression were recorded. The associations between the adherence and the clinical factors were analyzed by univariate and multivariate logistic regression. There were 204 patients included, of which 37 patients (18.14%) adhered to VF examinations. A total of 161 patients (78.9%) were diagnosed with open-angle glaucoma (OAG), 27 patients (13.2%) with angle closure glaucoma, and 16 patients (7.8%) with glaucoma suspect. Most of the participants (41.2%) had mild VF defect and had been prescribed with no more than 1 bottle of anti-glaucoma medication. In the multivariate analysis, diagnosis of glaucoma suspect (*P* = .02) and history of SLT (*P* = .04) were significantly associated with better adherence. Glaucoma severity and the announcement of glaucoma progression were not significantly associated with adherence to VF examination. The COVID-19 pandemic had greatly influenced the adherence to VF examination in glaucoma patients. This study demonstrated that patients with the diagnosis of glaucoma suspect and history of SLT were more likely to adhere to VF examinations even during the COVID-19 pandemic.

## 1. Introduction

Glaucoma is one of the leading causes of blindness worldwide.^[1]^ It is an optic neuropathy characterized by progressive loss of retinal nerve fiber layer accompanied with irreversible visual field loss.^[2]^ While reduction of intraocular pressure (IOP) has been proven to be a major influencing factor of glaucoma progression,^[3,4]^ most studies regarding the adherence of glaucoma patients only focused on adherence to anti-glaucoma medications.^[5–7]^ Adherence to follow-up examinations is crucial in monitoring glaucoma progression. However, there was limited literature discussing the adherence to follow-up visits and related examinations, especially in Asian countries.^[8]^ The American Academy of Ophthalmology recommended at least 2 follow-up visits per year and yearly visual field (VF) test in patients with primary open-angle glaucoma in the preferred practice pattern guidelines.^[9]^ Patients with poor adherence to follow-up examinations were more likely to have severer glaucomatous disease.^[10]^

The outbreak of Coronavirus disease 2019 (COVID-19) had a major impact on utilization of the healthcare services^[11]^ and the impact was greatest among low-income countries.^[11,12]^ Taiwan was one of the few countries that had initiated restrictions at the very early stage of the outbreak.^[13]^ Before first COVID-19 surge, there was almost 18 months of success in pandemic control in Taiwan, with <1300 cumulative cases until May 2021.^[14]^ Most residents were able to go to work and perform daily economic activities, including concerts, religious festivals and baseball games.^[14]^ After the outbreak in May 2021, an increase of 15,000 cases in 3 months were reported; meanwhile, over 100,000 confirmed cases were announced in the United States during this period.^[15]^ Despite a relatively low infection rate, Central Epidemic Command Center (CECC) in Taiwan raised the epidemic warning level for 2 major cities and strengthened related measures on May 15th, 2021.^[16]^ Many hospitals applied a strict COVID-19 screening policy for hospital visits during this period.

Although optical coherence tomography (OCT) provides a reliable and objective technique in glaucoma follow-up to measure peripapillary retinal nerve fiber layer and macular ganglion cell-inner plexiform layer thickness,^[17]^ the VF test still remains a standard role in glaucoma evaluation. With more follow-up examinations in the visual field, the progression speed would be more accurately estimated by the glaucoma progression analysis provided by Humphrey field analyzer (Carl Zeiss Meditec, Jena, Germany). However, there was a prominent reduction of visits in glaucoma clinics and a great portion of scheduled VF examinations were canceled by patients’ will. During this period, all patients had to provide a negative COVID-19 antigen test within 3 days from the scheduled VF examination according to our department regulation. The complicated process of antigen test and additional fee may have weakened the patients’ will to come back to complete the scheduled VF examination. Meanwhile, patients were anxious about getting infected by COVID-19 while visiting the hospital during this period. On the other hand, Pujari R. et al^[18]^ used a questionnaire to investigate the change in glaucoma patient experience during the COVID-19 pandemic, and they found that patients were more uncertain of their glaucoma care and more worried about losing vision from glaucoma. It is interesting and important to know the compliance of the glaucoma patients during this period. However, it is hard to determine the reason for glaucoma patients’ follow-ups, whether for glaucoma itself or for other reasons. The visual field examination is one of the most representative glaucoma examinations. Therefore, we aimed to analyze the impact of COVID-19 outbreak to the adherence to the scheduled VF examination during this short period and evaluated the possible associated factors regarding their adherence in a tertiary glaucoma referral center in New Taipei City in Taiwan.

## 2. Materials and methods

### 2.1. Study design and data collection

A retrospective chart review study was conducted in Far Eastern Memorial Hospital (FEMH). The study protocol was approved by the Ethics Committee of the institution (No:110278-E) and the study adhered to the tenets of the Declaration of Helsinki. The list of patients diagnosed with glaucoma and glaucoma suspect, who had scheduled VF examinations between May 17th, 2021, and July 12th, 2021 was investigated.

At FEMH, patients diagnosed with glaucoma were advised to have routine VF examinations every 3 to 6 months in adherence to American Academy of Ophthalmology preferred practice pattern guidelines.(9) During the study period, all patients had to provide a negative COVID antigen test within 3 days from the scheduled VF examination according to our department regulation. Those who adhered to this policy and received scheduled VF examination in the study period were defined as “adherence” and those who refused or delayed their VF examination over the study period were defined as “non-adherence.” Baseline demographic data and clinical characteristics were collected from the medical charts including type of glaucoma, duration of glaucoma, bottles of anti-glaucoma medications prescribed, IOP at the day of arranging VF examination, mean deviation (MD) of VF defect, and non-medical glaucoma treatment history including trabeculectomy and selective laser trabeculoplasty (SLT). VF tests were performed with a Humphrey field analyzer (Carl Zeiss Meditec, Jena, Germany) running the standard Swedish interactive threshold algorithm 30–2 program. The results were classified into 3 groups: mild defect (above −6 dB), moderate defect (−6 to −12 dB), and severe defect (<−12 dB). Duration of glaucoma was classified into 3 groups, which was <1 year, 1 to 5 years, and over 5 years. VF progression was defined by event-based analysis. Possible progression was announced when there were at least 3 locations that showed significant variability compared to baseline tests and repeated in the same points in at least 2 consecutive follow-up tests. Likely progression was announced when there were at least 3 locations that showed significant variability compared to baseline tests and repeated in the same points in at least 3 consecutive follow-up tests. “Possible progression” and “likely progression” in the latest VF examination before the scheduled VF examination in the study period were defined as VF progression in our analysis.^[19]^ Participants who had severe visual field defect (i.e., MD less than −20 dB) in the initial exam, were not eligible for event-based guided progression analysis and were defined as “severe VF defect.” Those participants who had only received <2 VF examinations were not eligible for event-based progression analysis, thus were classified as “insufficient examinations.”

Exclusion criteria were patients aged below 20 years or those with 1 eye legally blind. If bilateral eyes were glaucomatous, only the worse eye was analyzed in this study.

### 2.2. Statistical analysis

Statistical analysis was performed using SPSS 22.0 software (IBM Corporation, New York). Descriptive statistics such as the number and percentage for categorical data and the mean ± standard deviation for continuous data were used to present data distributions. Binary logistic regression was implemented to evaluate the association between each clinical characteristics and adherence to VF examination, controlling for age and gender. All variables associated with adherence in the univariable analysis with *P* value < .25 were included in multivariable regression.^[20]^ A value of *P* < .05 was considered statistically significant in multivariable analysis.

## 3. Results

During the study period, 205 patients with glaucoma had scheduled visual field examinations. One patient was excluded because he had one legally blind eye. Thirty-seven patients (18.14%) were classified into the adherence group, while 167 (81.86%) patients were classified into the non-adherence group. The mean age of participants was 54.22 ± 13.99 years. Of the 204 participants, 78.9% were diagnosed with open angle glaucoma (OAG), 13.2% were diagnosed with angle closure glaucoma, and 7.84% were diagnosed with glaucoma suspect. The majority (n = 165, 80.1%) of participants had been diagnosed with glaucoma for more than 1 year. Over half of the participants (n = 120, 58.8%) had been prescribed with no more than 1 bottle of anti-glaucoma medication. The mean IOP was 14.63 ± 3.90 mm Hg, and 95.6% of IOP was under 21 mm Hg. 5.4% of participants underwent trabeculectomy, while 16.7% underwent SLT. Around half of participants (n = 100, 49%) had mild VF defect (MD > −6 dB). 18.1% of participants had possible or likely progression in visual field progression analysis. The demographic data and clinical characteristics of study participants were listed in Tables 1 and 2.

**Table 1:** Demographic data of study participants (N = 204).

Variables	n	(%)
Age [mean ± SD] (yr old)	54.2 ± 14.0	
Gender [M:F]	120:84	
Glaucoma diagnosis		
OAG	161	(78.9)
ACG	27	(13.2)
Glaucoma suspect	16	(7.8)
Length of glaucoma history		
<1 yr	23	(11.3)
1–5 yr	98	(48.0)
>5yr	67	(32.9)
Glaucoma suspect	16	(7.8)
IOP*		
≤21 mm Hg	195	(95.6)
>21 mm Hg	9	(4.4)

ACG = angle closure glaucoma, IOP = intraocular pressure, OAG = open angle glaucoma.

* IOP was analyzed in the worse eye based on lower MD.

**Table 2:** Clinical characteristics of the study participants (N = 204).

Variables	n	(%)
AGM prescribed*		
≤1 bottle	120	(58.8)
>1 bottle	84	(41.2)
Operation history*		
Trabeculectomy	11	(5.4)
Selective trabeculoplasty	34	(16.7)
VF defect*,†		
≥−6 dB	100	(49.0)
−6 to −12 dB	37	(18.1)
<−12 dB	60	(29.4)
VF progression status*,‡		
No progression	92	(45.1)
Progression	37	(18.1)
Severe VF defect	26	(12.8)
Insufficient examinations	33	(16.2)

AGM, anti-glaucoma medication; VF, visual field; MD, mean deviation.

* Bottles of AGM prescribed, operation history, MD, VF progression were analyzed in the worse eye based on lower MD.

† Seven patients that had never took VF examinations were not included in the analysis of VF defect.

‡ Sixteen patients with glaucoma suspect were not included in the analysis of VF progression status.

Univariable analysis with odds ratio (OR) for above clinical characteristics were shown in Table 3. Significant clinical characteristics associated with adherence include type of glaucoma (*P* = .12), glaucoma progression (*P* = .05), and history of SLT (*P* = .2). In multivariable analysis, diagnosis of glaucoma suspect [OR = 5.06; 95% confidence interval (CI), 1.24–20.7, *P* = .02], and history of SLT [OR = 2.7; 95% CI, 4.04–6.98, *P* = .04] were significantly associated with greater adherence. Status of glaucoma progression was not significantly associated with adherence in multivariable analysis (Table 4).

**Table 3:** Univariable analysis of the clinical parameters associated with the adherence to scheduled visual field examination during the COVID-19 pandemic.

Variables	comparison	OR (95% CI)		*P* value
Glaucoma diagnosis				**.12**
	ACG vs OAG	1.31	(0.43–4.05)	0.64
	Suspect vs OAG	3.19	(1.05–9.76)	0.04
Length of glaucoma history				0.62
	1–5 yr vs ≤1 yr	0.68	(0.27–1.70)	
	>5 yr vs ≤1 yr	0.62	(0.23–1.71)	
AGM prescribed ≥ 1 bottle		1.44	(0.70–2.99)	.33
IOP ≥ 21 mm Hg		1.31	(0.41–4.21)	.65
History of trabeculectomy		1.01	(0.21–4.88)	.99
History of SLT		1.77	(0.74–4.21)	**.20**
VF MD				.91
	−6 to −12 vs ≥−6 dB	0.89	(0.32–2.47)	.82
	<−12 dB vs ≥−6 dB	1.14	(0.48–2.72)	.77
VF Progression status				**.05**
(Ref.: No progression)	Progression	0.46	(0.14–1.50)	.20
	Severe VF defect	0.33	(0.07–1.57)	.16
	Insufficient exams	2.03	(0.86–4.81)	.11

All variables in univariable analyses were adjusted with age and gender. Variables associated with adherence in the univariable analyses with *P* value < .25 were included in multivariable regression.

ACG = angle closure glaucoma, AGM = anti-glaucoma medication, IOP = intraocular pressure, MD = mean deviation, OAG = open angle glaucoma, VF = visual field.

**Table 4:** Multivariable analysis of the clinical parameters associated with the adherence to the scheduled visual field examination during COVID-19 pandemic.

Variables	comparison	OR (95% CI)		*P* value
Glaucoma diagnosis				.07
	ACG vs OAG	1.63	(0.50–5.28)	.42
	Suspect vs OAG	5.06	(1.24–20.70)	**.02**
History of SLT		2.70	(4.04–6.98)	**.04**
VF progression status				.11
(Ref.: No progression)	Progression	0.39	(0.12–1.31)	.13
	Severe VF defect	0.28	(0.06–1.39)	.12
	Insufficient exams	1.57	(0.60–4.11)	.36

All variables in multivariable analysis were adjusted with age and gender. *P* < .05 was considered statistically significant.

ACG = angle closure glaucoma, AGM = anti-glaucoma medication, OAG = open angle glaucoma, VF = visual field.

## 4. Discussion

COVID-19-related drop in volume of eye surgical procedures were reported.^[21]^ The pandemic has profoundly affected every social and medical field, even harmed the overall corneal transplant activity across Italy, with a reduction of nearly 60% as compared to the same period of 2019.^[22]^ Previous studies also reported impact on the adherence to lipid-lowering medications and biologics and reported that about 30 to 40 percent of patients failed to refill these medications after the first outbreak of COVID-19.^[23]^ Dramatic decrease of long-acting injectable antipsychotics prescriptions during this period was also reported.^[24]^ Researchers had proposed some theories for the adverse impact of COVID-19 pandemic on the adherence to medications. First, restrictive measures including limited physical distancing brought inconvenience when going out. Second, the elderly patients, who were more likely to have chronic conditions, were advised by every social media to stay at home. Also, mobilization of healthcare professionals to the COVID-19 frontline limited the accessibility of healthcare services.^[23]^ Reduced hospitalizations for chronic disease including heart failure and chronic obstructive pulmonary disease were also observed during COVID-19 pandemic.^[25]^ However, data about the reduction in ophthalmic examinations because of the lockdown restrictions were limited. This study in a tertiary referral center provides a comprehensive and in-depth overview of the impact of COVID-19 pandemic on the adherence to VF examinations, which was one of the most representative examinations in glaucoma patients. To the best of our knowledge, there was no study evaluating the impact of COVID-19 pandemic on glaucoma in the aspect of follow-up examinations instead of glaucoma treatment.

The COVID-19 outbreak in May 2021 in Taiwan resulted in major lockdown for almost 90 days. Meanwhile, FEMH adopted strict COVID-19 screening policies for all inpatient and outpatient departments. Patients who were scheduled for hospitalization or examinations in enclosed spaces with inadequate ventilation for over 15 minutes, including magnetic resonance imaging, hemodialysis and VF examinations…etc, were requested to take COVID-19 antigen test within 3 days. Although the screening policies were strict, which made the environment in the hospital were relative safer than the outside stores, the VF examination adherent rate was still very low. A total of 204 patients were enrolled, only 37 patients (18.14%) completed the VF examination. The fear of COVID-19 pandemic and inaccessibility of hospital-visit might result in the low adherence rate of VF examinations during this period. A systematic review revealed reduction by one third of healthcare utilization during COVID-19 pandemic.^[12]^ In one questionnaire-based study discussing the impact of COVID-19 in patients with glaucoma in South India, longer distance to hospital, using more than one anti-glaucoma medication, lack of awareness about glaucoma, and psychological affections were significantly associated with poor medication adherence.^[13]^ A more recent study revealed significant reduction of adherence rate of ocular hypotensive medication from 86.6% to 68% after COVID-19 pandemic outbreak in the United States.^[26]^ Their study provided more detailed insight on psychological effect on adherence in the COVID-19 pandemic. Lower psychometric measures of resilience, which resulted in lower levels of planful problem solving, was associated with poorer adherence.^[26]^ As far as we know, the factors we evaluated in our study were never discussed in previous studies, that we found the diagnosis of glaucoma suspect and history of SLT treatment were significantly associated with better adherence. It was interesting to find that the severity of glaucoma and the announcement of glaucoma progression were not significantly associated with the adherence to VF examination.

It has been well recognized that poor adherence to anti-glaucoma mediation, which usually refers to not regular using anti-glaucoma mediation, was associated with faster VF progression.^[27,28]^ However, few studies had evaluated the association between the adherence to follow-up examinations and VF progression status. Ung et al revealed that more severe VF loss and using more IOP-lowering medications were significant factors associated with poor follow-up visit adherence.^[10]^ In the Philadelphia glaucoma detection and treatment project, male gender, final diagnosis of glaucoma, and anti-glaucoma medications prescription were associated with greater adherence to follow-up examinations. This project was conducted before the COVID-19 pandemic and was performed in underserved urban communities.^[8]^ In our study, the severity of glaucoma and the announcement of glaucoma progression were not significantly associated with the adherence to VF examination. Two reasons might account for these findings. First, glaucoma was a chronic disease and if it was progressive, it was generally slowly progressive. Therefore, there would not be a sudden change in the severity of glaucoma, and patients with even severe glaucoma would not have better adherence to follow-up examination. Second, the glaucoma progression in this study was defined by the glaucoma progression analysis, which was usually too sensitive to be detected by the patients. These might explain the poor associations between the announcement of glaucoma progression and adherence to VF examination.

A history of SLT treatment was significantly associated with better adherence in our study. To the best of our knowledge, there was no literature studying the association of the adherence to glaucoma examination and history of the SLT treatment. Although previous studies suggested SLT can be an effective primary treatment in OAG eyes,^[29–31]^ IOP-lowering medications remained the first-line treatment for OAG in Taiwan. In FEMH, surgical and SLT treatment were only suggested to the patients with VF progression and unsatisfied IOP control or those intolerant to anti-glaucoma medication. However, when their IOP was controlled within low teens under topical medication, some patients hesitated and refused to receive further filtering surgery with the fear of surgical complications. They would rather choose SLT for further IOP control. Therefore, the awareness of unstable glaucoma control in patients who underwent SLT treatment might contribute to better adherence to VF examinations even during the period of COVID-19 pandemic. On the other hand, the eagerness to know if disease progression got controlled after the SLT treatment is the other reason for the higher adherence of VF examination in this group. On the contrary, history of trabeculectomy was not significantly associated with the adherence in our study. These patients usually had more severe VF defect and relatively poor visual acuity. However, they usually had more stable IOP after trabeculectomy and lower demand of anti-glaucoma medications after the trabeculectomy. This might result in careless attitude upon glaucoma follow-up examinations. Also, there were only 11 patients (5.4%) who underwent trabeculectomy in this study. The small sample size might have resulted in insufficient statistical power.

Diagnosis of glaucoma suspect was associated with better adherence to the VF examination in this study, compared to those who had confirmed diagnosis of glaucoma. Patients with glaucoma suspect were either referred from primary clinic for glaucoma evaluation or confirmation. The VF examination was crucial for glaucoma evaluation and deciding whether to commence anti-glaucoma medications. Moreover, VF examinations for patients were important for monitoring progression. Under National Health Insurance in Taiwan, it was easy to refill the anti-glaucoma medications at local pharmacies or local ophthalmological clinics. There was no crucial need of hospital visits, especially the tertiary teaching hospital only for refilling the medications during the COVID-19 pandemic. The other reason was that VF examination was not commonly available in local clinics. Therefore, even there were only 16 patients (7.8%) in the group of glaucoma suspect, we found significant association with better adherence to VF examination (*P* = .02) in the multivariable analysis. The eagerness to know whether one has glaucoma or not might result in less impact of COVID-19 pandemic on hospital utilization for patients with suspected glaucoma.

Our study has its limitations, including its retrospective design and relatively small sample size. Moreover, our study included patients from glaucoma suspect to advanced glaucoma. There were 2 circumstances not appropriate for analysis of VF progression, which were patients with insufficient examinations in the glaucoma suspect group, and those with severe VF defect in the advanced glaucoma group. The 2 populations accounted for 30% of the participants. The diversity of the status of VF progression may limit the statistic power. However, our study reflected the real-world data during the COVID-19 pandemic. Lastly, the outbreak of COVID-19 pandemic and the policy of the government was different in every country. This study was a single center study in Taiwan and the results may not directly apply to other countries. On the other hand, the special circumstances in this period with relatively low COVID-19 infection rate and tightened restrictions provided us a unique investigation of adherence to VF examination in the glaucoma patients. Further studies investigating delays in patient appointments of visual field examination with impacts on glaucomatous progression will further elucidate the real impacts that COVID-19 has brought to the glaucoma patients.

In conclusion, our study demonstrated the low adherence of scheduled VF examination in patients with glaucoma and glaucoma suspect during the COVID-19 pandemic. Patients with diagnosis of glaucoma suspect instead of glaucoma and history of SLT treatment were associated with greater adherence. Poor adherence to VF examinations may result in delay of diagnosing and detecting disease progression. The findings of our study may shed light on the importance of adherence to VF examination and awareness the adverse impacts of disease monitoring during COVID-19 pandemic.

## Author contributions

**Conceptualization:** Pei-Yao Chang, Yu-Han Wang.

**Data curation:** Yu-Han Wang.

**Formal analysis:** Yu-Han Wang, Jiun-Yi Wang.

**Investigation:** Yu-Han Wang.

**Methodology:** Pei-Yao Chang, Yu-Han Wang.

**Project administration:** Pei-Yao Chang, Yu-Han Wang.

**Resources:** Yu-Han Wang.

**Software:** Yu-Han Wang.

**Supervision:** Pei-Yao Chang, Yu-Han Wang, Jia-Kang Wang.

**Validation:** Yu-Han Wang.

**Visualization:** Yu-Han Wang, Jiun-Yi Wang.

**Writing – original draft:** Yu-Han Wang.

**Writing – review & editing:** Pei-Yao Chang.
